# The Reliability of Neurological Measurement in the Vastus Medialis: Implications for Research and Practice

**DOI:** 10.3389/fpsyg.2018.01857

**Published:** 2018-10-01

**Authors:** Hans Leung, Christopher Latella, Séverine Lamon, Ashlee M. Hendy

**Affiliations:** School of Exercise and Nutrition Sciences, Institute for Physical Activity and Nutrition (IPAN), Deakin University, Geelong, VIC, Australia

**Keywords:** transcranial magnetic stimulation, corticospinal, H-reflex, leg extensors, inter-session

## Abstract

The integrity of the corticomotor pathway is paramount in the optimal functioning of skeletal muscle. However, variability of neurophysiological assessment via peripheral nerve and transcranial magnetic stimulation can render interpretation difficult. Seldom evidence exists regarding the reliability of such measurements in the leg extensors, which have important locomotive and functional roles. This study aimed to assess the test-retest reliability of peripheral, corticospinal and intracortical responses in the vastus medialis. Transcranial magnetic and direct current electrical nerve stimulation were delivered to sixteen healthy young adults (8M and 8F) on two separate occasions. The Hoffmann reflex, maximal compound wave, motor evoked potential, corticospinal silent period, intracortical facilitation, and short-interval intracortical inhibition were recorded from the vastus medialis at rest, and during controlled submaximal voluntary contraction. Relative reliability was quantified using intra-class correlation coefficient (ICC2,1). Absolute reliability was quantified using standard error of measurement (SEm) and minimal detectable change (MDC). Corticospinal silent period, corticospinal silent period/motor evoked potential ratio, active motor evoked potential, maximal Hoffman reflex, and passive short-interval intracortical inhibition demonstrated “good to excellent” relative reliability (ICC ≥ 0.643). Intracortical facilitation demonstrated the lowest relative reliability (ICC = 0.420–0.908). Corticospinal silent period displayed the lowest absolute reliability (SEm ≤ 18.68%). Good reliability of the maximal compound wave, Hoffman reflex, motor evoked potential, and corticospinal silent period allow for reliable neurological evaluation of peripheral and corticospinal pathways in the vastus medialis. Future research should investigate reliability of the intracortical (short-interval intracortical inhibition and intracortical facilitation) measures by using different paired-pulse stimulus parameters. These findings hold important implications for neurophysiological assessment conducted in the leg extensor group.

## Introduction

The integrity of the human motor system is essential in the development of muscular force, power and overall physical function. Stimulation of intracortical and corticospinal pathways is commonly used to provide insight into the behavior of excitatory and inhibitory networks underpinning motor control of movement. In particular, the musculature of the lower limbs play an important role in functional and performance tasks, locomotion and knee joint integrity (Avramidis et al., 2003; Peeler and Anderson, 2007). Therefore, the reliable assessment of the motor pathway associated with the lower limbs is paramount in clinical, research and human performance settings. In this research, we investigated the lower-limb muscle VM for two reasons; (1) the VM generates the most consistent H-reflex response compared to the VL and rectus femoris (Kameyama et al., 1989) and (2) the VM plays an important role in the function and injury prevention of the knee. The VM has a crucial influence on gait performance and motor control of the knee (Avramidis et al., 2003), and its weakness is viewed as a contributing factor to patellofemoral joint dysfunction (Peeler and Anderson, 2007). The delayed onset of EMG activity of the VM relative to the VL, which indicate neurological dysfunction, is common in patients with patellofemoral pain syndrome (Cowan et al., 2001). Furthermore, the VM tends to atrophy to a greater extent than the other quadriceps muscles during disuse (Appell, 1990).

Peripheral nerve stimulation provides insight into the pre- or post-synaptic excitability of Ia afferent reflex pathways and/or the excitability of lower motor neurons (Pierrot-Deseilligny and Mazevet, 2000). This has been used to investigate musculoskeletal injury (Hopkins and Palmieri, 2004), neurological conditions (Braddom and Johnson, 1974), exercise based adaptations (Earles et al., 2002), and fatigue (Latella et al., 2017). The reliability of the H-reflex has generally been reported in the upper limbs (Miller et al., 1995; Bodofsky, 1999) and ankle flexors (Hopkins et al., 2000; Palmieri et al., 2002). There is, however, some debate around the reliability of these measures in the VM. Previously, “excellent” within-subject reliability has been reported in the VM across six subsequent days (Hopkins and Wagie, 2003), whilst others reported poor reliability due to high within-subject variation (Doguet and Jubeau, 2014). Calder et al. (2005) reported “excellent” reliability of the MMAX in the elbow flexors. Recently, Nuzzo et al. (2016) suggested a posture-dependent effect on MMAX, questioning the reliability of its use in various research protocols. In the lower limbs, Latella et al. (2017) reported “good” reliability of MMAX, recorded from the rectus femoris. However, this has not been comprehensively investigated in the VM.

Transcranial magnetic stimulation provides a non-invasive assessment of the entire motor pathway and is used extensively in clinical and research settings (Sacco et al., 1997). TMS can quantify temporal and spatial information (i.e., cortical, corticospinal, and spinal) with single- and paired-pulse stimulation techniques. Despite its widespread use, the evoked responses demonstrate moderate to large intra- and inter-tester and -participant variability (Orth and Rothwell, 2004; Cavaleri et al., 2017). In particular, factors such as circadian rhythm (Sale et al., 2007), age (Remaud et al., 2014; Mouthon et al., 2016), ovarian hormones (Inghilleri et al., 2004), fatigue (Taylor et al., 2006), and training status (Pearcey et al., 2014) can influence corticospinal excitability. Orth and Rothwell (2004) have previously reported that the inter-subject variation of the CSP of an intrinsic hand muscle is high, but is reduced when expressed as a ratio of the MEP. More commonly, the MEP/MMAX ratio is reported as an alternative means of normalization to reduce inter-subject variability. Further consideration must also be given to the influence of stimulation during passive rest or active contraction, with variability generally decreasing during muscular activation (Thomas et al., 2016). The reliability of the TMS has been well reported in the upper limbs (Maeda et al., 2002; Wassermann, 2002; Kamen, 2004; Ngomo et al., 2012), but is less common in the lower limbs. To our knowledge, only a handful of authors have established MEP reliability in the VM during isometric contraction (Luc et al., 2014; Temesi et al., 2017), whilst to our knowledge, passive MEP reliability has not been investigated. Additionally, no studies have concurrently investigated peripheral nerve and corticospinal measures in the VM. In paired-pulse protocols, Thomas et al. (2016) and Temesi et al. (2017) have reported “good” reliability of short and long interval cortical inhibition (SICI and LICI) in the VM. Although the authors also reported “good” reliability of ICF, this has not always been reported (Latella et al., 2017). Given that it is increasingly common for neurological studies to use single- and paired-pulse TMS as their outcome measures (Pearcey et al., 2014; Remaud et al., 2014; Hunter et al., 2016; Latella et al., 2016, 2017; Mouthon et al., 2016), the reliability of these measures should be established. Traditional TMS neurological study protocols have generally been developed for studies investigating intrinsic hand muscles, not for the lower limb. In particular, the disparate functional roles, fiber type and architectural composition of the muscles within the leg extensor group suggest that further research is warranted to elaborate on previous findings.

The aim of this study was to investigate the reliability of the neurophysiological responses in the VM. In particular, we aim to assess peripheral (H-reflex and M-wave), corticospinal (MEP and CSP) and intracortical (SICI and ICF) measures evoked from peripheral nerve and TMS. The results of this study will help inform future neurophysiological studies investigating the VM and will hold important implications for future lower limb rehabilitation research and clinical practice.

## Materials and Methods

### Participants

Sixteen (8 male, age: 25.88 ± 3.83 years, 8 female, age: 23.13 ± 2.95 years) healthy participants participated in the study. Written informed consent was obtained for each participant prior to the start of the study. Prior to TMS, all participants were screened using a TMS safety questionnaire to exclude potential participants with contraindications to neurological testing (Rossi, 2009). All procedures used in this study were approved by the Deakin University Human Research Ethics Committee (2017-023) and conducted to the standards set by the Declaration of Helsinki.

### Experimental Protocol

Participants performed 2 × 1 h testing sessions (Day 1 and Day 2), separated by a period of 7–10 days. All sessions were conducted at the same time of the day. Each session involved MVIC and, peripheral and transcranial nerve stimulation. The procedures for the pre and post testing were identical.

### Maximal Voluntary Isometric Contraction

Maximal torque of the leg extensors was measured during a 3 s MVIC. Participants performed three MVIC trials (2 s ramp up and 3 s maximal contraction), separated by a 60 s rest period. Trials were conducted with the participants seated upright on an isokinetic dynamometer (Biodex system 4 pro, Biodex medical systems, United States). An immovable leg extension arm was secured approximately 2 cm above the medial malleolus (Krishnan et al., 2011) and the ankle of the right foot strapped in place. The hip was positioned at 90o of flexion with a 60o flexion angle of the right knee. Strong verbal encouragement was provided to the participant by the researchers. The maximal peak torque (Nm) of the three trials was recorded as the MVIC.

### Surface Electromyography

Surface electromyography activity was recorded from the VM muscle in the right leg using bipolar Ag-AgCl electrodes. Two electrodes positioned 20 mm apart were placed over the muscle belly of the VM at the 80% mark of the line between the anterior spina iliaca superior and the joint space in front of the anterior border of the medial ligament (Hermens et al., 2000; Fratini et al., 2009). The participants’ skin was shaved and cleaned with an isopropyl alcohol swab prior to electrode placement to ensure a clear signal was obtained. Surface electromyography activity signals were analyzed offline using PowerLab 4/35 (ADinstruments, Australia).

### Peripheral Nerve Stimulation

**FIGURE 1:**
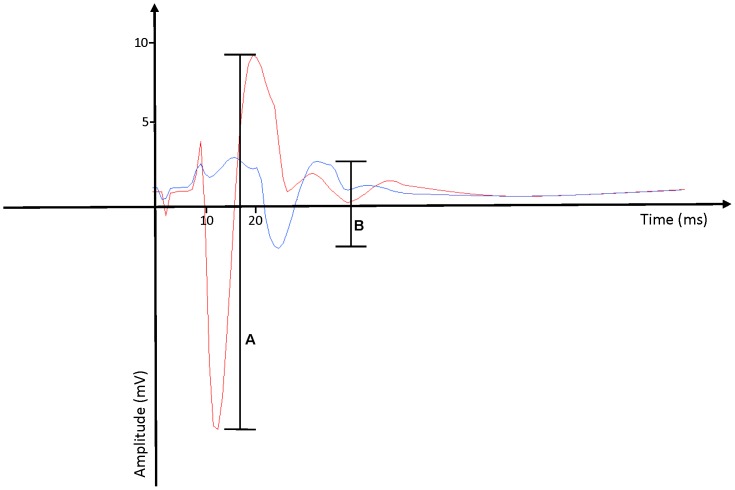
Examples of **(A)** MMAX response and **(B)** H-reflex response, as measured by surface electromyography recordings. MMAX response typically occurs at around the 9 ms mark. H-reflex response typically occurs at around the 15–20 ms mark.

The H-reflex and M-wave were evoked via electrical stimulation of the right femoral nerve using a constant current stimulator (Digitimer, Hertfordshire, SDR Scientific). To maintain a consistent posture (Cecen et al., 2018), participants laid supine, with their leg resting passively and in a slightly flexed, supported position. Nerve stimulation (pulse width 1 ms) was delivered with bipolar electrodes placed over the femoral triangle approximately 3–5 cm below the inguinal ligament along the right inguinal fold (Doguet and Jubeau, 2014). The stimulus intensity was increased until the peak-to-peak amplitude of the M-wave became saturated (MMAX). **Figure 1** depicts an example of a MMAX obtained from one of our participants, represented by **Figure 1A**. The stimulus intensity was then increased by a further 20% to ensure a supramaximal stimulus was delivered each time. The same procedure was applied for active MMAX measures, but with participants flexing their right knee at a 60o angle (full knee extension equates to 0o), and contracting at 10% of MVIC.

To assess the H-reflex, the stimulus intensity was initially reduced to a point where no response was observed, then increased at increments of 2.5 mA until the largest peak-to-peak response. Offline examination of the wave-form was conducted manually with a cursor, and the largest amplitude was reported as the H_(MAX)_. This value was also used to determine the H_(MAX)_:M_(MAX)_ ratio (Doguet and Jubeau, 2014). The H-reflex was observed approximately 15–25 ms after stimulation (Palmieri et al., 2004). **Figure 1** depicts an example of a HMAX response from one of our participants, represented by **Figure 1B**. Successive stimuli were separated by a 20 s inter-stimulus interval to minimize any potentiation and/or fatigue (Hopkins and Wagie, 2003).

### Transcranial Magnetic Stimulation

All TMS was delivered with the participant seated upright on the isokinetic dynamometer. For passive conditions, participants were instructed to relax their right leg in the seated position, whilst for active measures, participants were instructed to hold at a steady 10% MVIC using real time visual feedback. Both conditions were conducted at 60° of flexion. TMS was delivered over the motor cortex (M1) using a 110 mm concave double-cone coil (Magstim Co., United Kingdom) attached to a BiStim 2002 magnetic stimulator (Magstim Co., United Kingdom). The coil was positioned horizontally in an anterior–posterior direction while delivering the stimulus. Participants wore a tight fitting cap (EasyCap, Germany), positioned with reference to the nasion-inion and interaural lines to ensure intra- and inter-test consistency of the coil placement. To locate the optimal site, stimuli were delivered over various points along the M1. The optimal site was the location on the M1 that evoked the greatest MEP amplitude in the right VM. This spot was then marked down on the cap and used for further testing. The resting motor threshold (RMT) and active motor threshold (AMT) were defined as the lowest stimulus intensity required to elicit an MEP of at least 50 or 200 μV, respectively, in at least six out of 10 trials (Rothwell et al., 1999). Corticospinal excitability was determined by the peak-to-peak amplitude of the single pulse waveform at a stimulus intensity of 1.2 times AMT and RMT from 10 trials (Chen, 2000). The CSP was calculated as the duration in ms from the onset of the MEP to the return of pre-stimulation surface electromyography activity level.

Paired-pulse stimulation was used to assess intracortical inhibition and facilitation. Ten pairs of conditioning- and test-pulses were delivered at 80% and 120% of RMT, respectively. The inter-stimulus interval was set at 3 and 12 ms for SICI and ICF, respectively (Wassermann, 2008; Rossini et al., 2015). Passive conditions were investigated for both SICI and ICF and an additional active condition was investigated for SICI. Both SICI and ICF were expressed as a percentage of the average single-pulse MEP (Wassermann, 2008; Rossini et al., 2015).

### Statistical Analysis

All data was analyzed using IBM SPSS Statistics v.25 (IBM, United States). Test-retest reliability was then analyzed for each outcome measure (MVIC, MEP, MEP/MMAX, CSP, CSP/MEP, ICF and SICI, and MMAX and HMAX). Relative overall reliability was assessed by calculating the two-way mixed effects, absolute agreement, single measurement ICC2,1 (Koo and Li, 2016). ICCs were classified as “poor” (<0.40), “fair” (0.40–0.59), “good” (0.60–0.74), and “excellent” (≥0.75) (Temesi et al., 2017). The within participant coefficient of variation (CV) was expressed as the average of each individual’s CV, a percentage derived from the formula (SD/Mean) × 100. CV values ≤10% indicate low variability. Absolute reliability was calculated to establish the variability of repeated measurements (Atkinson and Nevill, 1998) using the *Standard error of measurement* (*SEm*) = *SD*$\sqrt{1-\mathrm{ICC}}$ (Weir, 2005). The MDC was calculated via the formulae *MDC* = *SEM* × 1.96 × $\sqrt{2}$, to determine to minimum difference required between trials for the change to be considered real (Weir, 2005). Bland–Altman plots were used to visualize the agreement between the two trials. 95% limits of agreement (LOA) was calculated via the formulae *x* ± 1.96 × *SD*, where x is the mean difference between the two trials, and SD is the standard deviation of differences of the trials. The smaller the range of the LOAs, the better the agreement is.

## Results

### Maximal Voluntary Isometric Contraction

**Table 1** displays the reliability of the MVIC measurements. The relative reliability (ICC = 0.945, 95% CI [0.852–0.980]) was “excellent.” The SEm, CV, and MDC were 19.8, 8.30%, and 55.0, respectively.

**Table 1:** Reliability data for all peripheral measures across two testing sessions.

	Day l	Day 2	ICC	95% CI	SEm (%)	CV%	MDC
MVIC(N)	163.13 ± 58.66	165.88 ± 60.84	0.945	0.852–0.980	19.8 (12.05)	8.30	55.0
Passive M_MAX_ (mV)	5.85 ± 2.65	6.51 ± 2.83	0.843	0.599–0.943	1.55 (25.02)	14.93	4.29
Active M_MAX_ (mV)	6.52 ± 3.58	6.39 ± 3.80	0.896	0.728–0.962	1.67(25.75)	19.43	4.62
H_MAX_ (mV)	1.75 ± 1.20	1.88 ± 1.07	0.803	0.528–0.926	0.715 (39.36)	24.99	1.98
H:M Ratio (%)	35.27 ± 22.78	34.65 ± 23.18	0.860	0.643–0.949	0.122 (34.79)	23.99	0.337

*MVIC, maximal voluntary isometric contraction; MMAX, maximal compound wave; HMAX, maximal Hoffman reflex; ICC, intra-class correlation; CI, confidence interval; SEm, standard error of measurement; CV, coefficient of variation; MDC, minimal detectable change; N, newtons; and mV, millivolts.*

### Peripheral Nerve Stimulation

**FIGURE 2:**
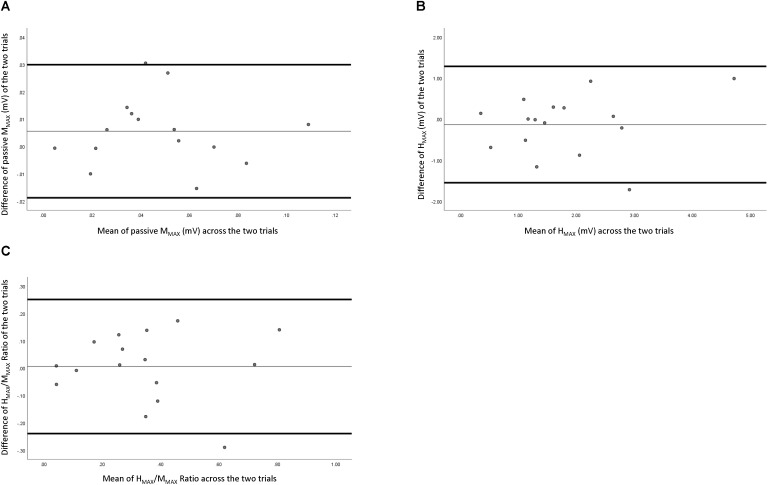
Bland–Altman plot of peripheral measures across the two testing sessions. **(A)** Resting maximal compound wave, **(B)** maximal Hoffman reflex, and **(C)** maximal Hoffman reflex normalized with maximal compound wave.

Reliability data of peripheral nerve measures are presented in **Table 1**. **Figure 2** depicts the comparison of individual measurements across the two trials. Passive MMAX had “fair to excellent” relative reliability (ICC = 0.599–0.943), a SEm of 1.55, a CV of 14.93%, and a MDC of 4.29. Active MMAX had “good to excellent” relative reliability (ICC = 0.728–0.962), a SEm of 1.67, a CV of 19.43%, and a MDC of 4.62. HMAX had “fair to excellent” relative reliability (ICC = 0.528–0.926), a SEm of 0.715, a CV of 24.99%, and a MDC of 1.98. HMAX:MMAX (H:M) ratio had “good to excellent” relative reliability (ICC = 0.643–0.949), a SEm of 0.122, a CV of 23.99%, and a MDC of 0.337.

### Single-Pulse Transcranial Stimulation

**FIGURE 3:**
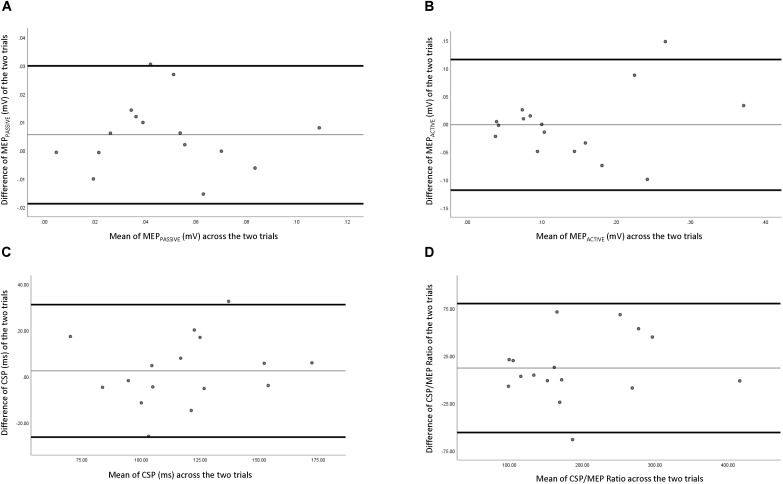
Bland–Altman plot of corticospinal measures across two testing sessions. **(A)** Passive motor evoked potential, **(B)** active motor evoked potential, **(C)** corticospinal silent period, and **(D)** ratio of corticospinal silent period and active motor evoked potential.

Reliability data of corticospinal measures are presented in **Table 2**. **Figure 3** depicts the comparison of individual measurements across the two trials. MEPpassive had “good to excellent” relative reliability (ICC = 0.691–0.961), a SEm of 0.0132, a CV of 17.45%, and MDC of 0.0365. MEPactive had “fair to excellent” relative reliability (ICC = 0.575–0.937), a SEm of 0.0581, a CV of 17.45%, and a MDC of 0.161. CSP had “good to excellent” relative reliability (ICC = 0.663–0.951), SEm of 14.4, a CV of 7.24%, and a MDC of 40.0. CSP/MEP ratio had “excellent” relative reliability (ICC = 0.793–0.972), a SEm of 35.8,a CV of 9.68%, and a MDC of 99.1.

**Table 2:** Reliability data for all corticospinal and intracortical measures across two testing sessions.

	Day 1	Day 2	ICC	95% CI	SEm (%)	CV *(%)*	MDC
MEP_passive_	0.050 ± 0.03	0.045 ± 0.03	0.887	0.691–0.961	0.0132 (27.82)	17.45	0.0365
MEP_active_	0.14 ± 0.11	0.14 ± 0.09	0.830	0.575–0.937	0.0581 (41.52)	19.69	0.161
CSP (ms)	119.26 ± 32.73	116.73 ± 31.27	0.866	0.663–0.951	14.4 (12.24)	7.24	40.0
CSP/MEP	197.53 ± 116.85	1S5.41 ± 108.65	0.921	0.793–0.972	35.8 (18.67)	9.68	99.1
SICI_passive_	0.19 ± 0.14	0.20 ± 0.15	0.874	0666–0.956	0.0724 (36.60)	22.85	0.201
SICI_active_	0.40 ± 0.16	0.41 ± 0.17	0.783	0480–0.919	0.111 (27.15)	17.14	0.308
ICF	1.60 ± 1.03	1.39 ± 0.68	0.751	0.420–0.908	0.620 (41.53)	19.22	1.72

*MEP, motor evoked potential; CSP, cortical silent period; SICI, short-interval intracortical inhibition; ICF, intracortical facilitation; ICC, intra-class correlation; CI, confidence interval; SEm, standard error of measurement; CV, coefficient of variation; and MDC, minimal detectable change.*

### Paired-Pulse Transcranial Stimulation

**FIGURE 4:**
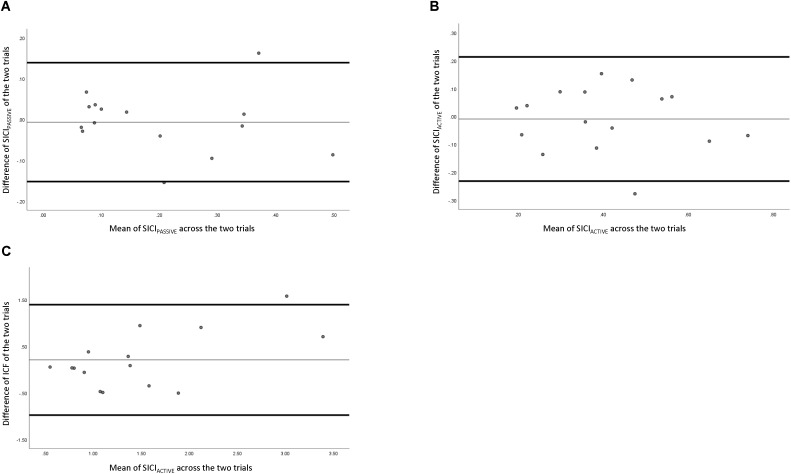
Bland–Altman plot of intracortical measures across two testing sessions. **(A)** Passive short-interval intracortical inhibition, **(B)** active short intracortical inhibition, and **(C)** intracortical facilitation.

Reliability data of intra-cortical measures are presented in **Table 2**. **Figure 4** depicts the comparison of individual measurements across the two trials. SICIpassive had “good to excellent” relative reliability (ICC = 0.666–0.956), SEm of 0.0724, a CV of 22.85%, and a MDC of 0.201. SICIactive had “fair to excellent” relative reliability (ICC = 0.480–0.919), a SEm of 0.111, a CV of 17.14%, and a MDC of 0.308. ICF had “fair to excellent” relative reliability (ICC = 0.420–0.908), a SEm of 0.620, a CV of 19.22%, and a MDC of 1.72.

## Discussion

The aim of this study was to investigate the reliability of transcranial magnetic and peripheral nerve stimulation outcome measures in the VM. Specifically, we assessed the test–retest relative and absolute reliability of peripheral (MVIC, MMAX, and H-reflex), corticospinal (MEP and CSP), and cortical (SICI and ICF) responses. Our findings suggest that the peripheral nerve measures are the most reliable. Corticospinal responses, specifically MEPpassive, CSP, and CSP/MEP, demonstrated “good to excellent” relative reliability whilst active MEP demonstrated the lowest reliability. For intracortical responses, the relative reliability of SICIpassive was “good to excellent.” In contrast, ICF and SICIactive demonstrated “poor to excellent” reliability. The findings of this investigation suggest that non-invasive cortical and peripheral nerve stimulation of the VM offer reasonable intra-participant and inter-session reliability in most measures of neurophysiological function.

### Peripheral Nerve and Spinal Excitability

The findings demonstrated that the spinal reflex afferent and efferent pathways can be reliably measured in the VM. Our results showed “good to excellent” intra- and inter-participant relative reliability for active MMAX and H:M ratio. These findings are similar to those of Hopkins and Wagie (2003), who showed acceptable reliability of the VM H-reflex over six testing sessions. It is worth noting that H:M ratio was more reliable than HMAX, indicating the importance to normalize HMAX with MMAX when evaluating spinal excitability. In terms of absolute reliability, H-reflex measurements displayed a higher measurement error (SEm > 30%) compared to MMAX measurements (SEm < 26%). However, the within-subject variation for the resting H-reflex (CV = 24.99%) in our study is considerably lower than those reported by Doguet and Jubeau (2014) (CV ≥ 52.2%). This large discrepancy between the studies may be due to the different interstimulus intervals used: 20 s versus the 10 s interval used by Doguet and Jubeau (2014). The cumulative effects of short repeated stimulation potentially influences monosynaptic reflex activity, due to fatigue or potentiation. Given that fatigue is unlikely to have developed in either condition, the difference may be attributed to a potentiation effect or the longer muscle length (75 degrees of knee flexion) used by Doguet and Jubeau (2014). In particular, this lengthened position can induce spinal excitability disturbances due to increased muscle spindle firing (Duclay and Martin, 2005). Therefore, we conclude that the H-reflex and MMAX can provide a reliable measurement of monosynaptic afferent reflex pathways and motor pool activation when external factors are carefully controlled for. Researchers should therefore consider factors that may influence spinal and lower motor neuron excitability when conducting neurophysiological assessment of the VM. These factors include participants’ posture, knee flexion angle, and interstimulus interval used. To allow for feasible session duration and a broad spectrum of neurophysiological assessments (peripheral, corticospinal, and intracortical), we did not collect full input-output curves for any of the measures. See **Supplementary Data Sheet 1** for example of modified input-output curve and raw H-reflex traces elicited by peripheral nerve stimulation. This must be acknowledged as a limitation, as previous work has highlighted the value of investigating the entire H-reflex and M-wave recruitment curve (Brinkworth et al., 2007; Tucker et al., 2005). We recommend that researchers consider factors such as session duration, participant burden, and expected longevity of the neuromodulatory intervention, alongside the relative and absolute reliability of the measures when determining the appropriate testing protocols.

### Corticospinal Excitability and Inhibition

The current research demonstrated “good to excellent” relative reliability of the MEPpassive, CSP, and CSP/MEP ratio in the VM. The MEPpassive (ICC = 0.691–0.961) proved to be a more reliable measure compared to the “fair to excellent” relative reliability of MEPactive (ICC = 0.575–0.937). This is in line with Ngomo et al. (2012), who reported slightly higher relative reliability in the resting muscle. This may be caused by subtle differences in motor unit recruitment during the active condition, despite dynamometer force output appearing constant. For example, minor alterations in joint angle, limb positioning and factors such as muscle fatigue may mean that motor unit recruitment varies, despite force output appearing steady. In terms of absolute reliability, MEPpassive also displayed a lower measurement error than MEPactive. The MEPactive result was similar to that of Latella et al. (2017) who also recorded responses in the leg extensors. Interestingly, this is contrary to previous work by O’Leary et al. (2015) and Luc et al. (2014), who have concluded “excellent” reliability of the active MEP in the knee extensors. It is worth noting that both these studies used a stimulus intensity that exceeded 120% of AMT. Ngomo et al. (2012) and Luc et al. (2014) suggest that higher stimulation intensities may improve the reliability of active MEPs, with Luc et al. (2014) finding 130 and 140% of AMT to be more reliable compared to 105–120% of AMT. In addition, the CSP and CSP/MEP ratio showed “good to excellent” (ICC = 0.663–0.951) and “excellent” (ICC = 0.793–0.972) reliability, respectively, which is consistent with the findings of O’Leary et al. (2015) in the VL. The CSP and CSP/MEP ratio also demonstrated better absolute reliability (SEm < 20%) compared with the MEP responses. CSP is partly mediated by the inhibition of gamma-aminobutyric acidB receptors in both spinal and cortical circuits (Lefaucheur et al., 2008). Although the reliability of the CSP indicates that corticospinal inhibition can be accurately measured, the CSP/MEP ratio may offer a better insight into the relationship between excitability and inhibition (Orth and Rothwell, 2004). Our results suggest that CSP and CSP/MEP ratio are reliable outcome measures when evaluating corticospinal inhibition. Compared with MEPactive, MEPpassive appears to be a more reliable outcome measure when evaluating corticospinal excitability in the VM.

### Intracortical Facilitation and Inhibition

The results demonstrated “good to excellent” and “fair to excellent” relative reliability of the SICIpassive (ICC = 0.666–0.956) and SICIactive (ICC = 0.480–0.919), respectively. To our knowledge, no study has investigated the reliability of SICIpassive in the VM, potentially due to the difficulty in obtaining large resting MEP responses in the lower limbs. Our results are in agreement with previous work in the upper limb, which suggested SICI should be assessed under passive conditions in the first dorsal interosseous (Ngomo et al., 2012). This may be partially explained by the fact that muscle activity causes a reduction in intracortical inhibition, and increased activation of facilitatory circuits (Ortu et al., 2008). In terms of absolute reliability, SICIactive displayed a lower level of variability compared with SICIpassive. O’Leary et al. (2015) suggested that SICIactive may be less variable than SICIpassive due to the reduced attentional and somatosensory influences. Using a stronger conditioning stimulus (90% of AMT) increased reliability of SICIactive (O’Leary et al., 2015). This is also supported by (Temesi et al., 2017), which reported “excellent” relative reliability for SICIactive in the VM with a conditioning stimulus of 90% of AMT. Using a weaker conditioning stimulus may induce less inhibitory interneurons compared to a stronger conditioning stimulus, leading to a less consistent SICI response (O’Leary et al., 2015). Temesi et al. (2017) also reported “excellent” reliability for SICIpassive, but poor reliability for SICIactive in the hand. Although at this stage it is difficult to pinpoint the mechanisms responsible for the differences between the upper and lower limbs, this finding may be at least in part due to the difference in functional connectivity. For example in locomotion, reciprocal inhibition of the opposite homologous muscle is required, therefore inhibitory circuitry may be more prominent in the motor neurons supplying the lower limbs (Chiou et al., 2013; Hendy et al., 2017). As the functional roles and inhibitory responses between the upper and lower limbs are different, testing parameters may need to be modified for specific muscle groups to obtain the most reliable results. Our results, along with previous reports, suggest that SICI with a conditioning stimulus of 90% of AMT demonstrate the greatest reliability when evaluating intracortical inhibitory mechanisms in the VM. Finally, our findings demonstrated that the ICF was the least reliable (ICC = 0.420–0.908) out of all measures. Similar to previous studies, ICF was less reliable than both resting and active SICI (Maeda et al., 2002; Fleming et al., 2012; Du et al., 2014; O’Leary et al., 2015), despite methodological differences. For instance, O’Leary et al. (2015) used different conditioning stimulus intensities for their SICI (70, 80, and 90%) and ICF (90, 100, and 110%) measures when establishing reliability. It is known that both SICI and ICF are affected by GABA_A_ receptors (Di Lazzaro et al., 2000), however, ICF is also affected by changes in NMDA receptor sensitivity (Hermsen et al., 2016). Thus, it could be speculated that the lower relative reliability of ICF might be due to mechanisms involving NMDA, however, confirmation from pharmaceutical studies is required. Further research to compare the reliability of different conditioning and test stimulus intensities to quantify SICI and ICF is required to confirm the most reliable protocol when evaluating intracortical excitabitory and inhibitory mechanisms in the VM.

While we report that collectively the corticospinal and intracortical measures demonstrated “good” reliability, it is unclear from this study whether adjusting the conditioning and testing stimulus intensities or ISI may have altered the results. A recent study found that using the average of multiple ISIs led to higher relative and absolute reliability for SICI in the hand (Matamala et al., 2018). Additionally, the value of the ICC in reliability studies must be interpreted with caution. While many have tried to place levels to evaluate the magnitude of ICC, it is not possible to set a universal standard of what constitutes a “good” or “excellent” ICC (Charter and Feldt, 2001). The magnitude of the ICC ultimately depends on between-subjects variability. Assuming the same within-subject variabilities, the higher the between-subjects variability, the larger the ICC value (Weir, 2005). In this study, all the neurological measures exhibit a large degree of between-subjects variability, which elevates the ICC. To account for this, we used SEm, MDC, and CV, which are measures of absolute reliability (Weir, 2005). Future test-retest reliability studies in the field should report both absolute and relative reliability measures, and seek to develop rigorous guidelines for absolute reliability. This information will contribute to establish a valid, systematic approach to investigating neurophysiological changes associated with human performance and rehabilitation studies.

## Conclusion

Collectively the results suggest that H:M Ratio, MEPpassive, CSP, and CSP/MEP ratio can provide reliable assessment of reflex pathways, excitability and inhibition in the VM. ICF in particular should be used with caution, especially when translating findings into neurological outcomes, such as determining mechanisms underpinning acute or adaptive changes in the leg extensors following interventions. In order to establish the most reliable neurological testing protocols, future studies should compare the reliability of peripheral, corticospinal and intracortical measures for different muscle groups, using various stimulus intensities and ISIs. Protocols need to be established and individualized for the VM, since initial protocols developed in the upper limb may not be optimal for lower limb studies.

## Author Contributions

AH and SL developed the conceptual basis and experimental design for this study. HL conducted data collection. HL and CL carried out data analysis, interpretation of results, and manuscript preparation. AH, SL, and CL provided supervision and wrote feedback throughout the research process.

## Conflict of Interest Statement

The authors declare that the research was conducted in the absence of any commercial or financial relationships that could be construed as a potential conflict of interest.

## Abbreviations

CSP: corticospinal silent period
GABAA: gamma-aminobutyric acid A
HMAX: maximal Hoffman reflex
H-reflex: Hoffmann reflex
ICC: intra-class correlation coefficient
ICF: intracortical facilitation
MDC: minimal detectable change
MEP: motor evoked potential
MMAX: maximal compound wave
MVIC: maximal voluntary isometric contraction
NMDA: N-methyl-d-aspartate
SEm: standard error of measurement
SICI: short-interval intracortical inhibition
TMS: transcranial magnetic stimulation
VM: vastus medialis.
