# Supplementary material for: The Reliability of Neurological Measurement in the Vastus Medialis: Implications for Research and Practice
Source: Front Psychol. 2018 Oct 1;9:1857. doi: 10.3389/fpsyg.2018.01857 (PMC6174212; doi:10.3389/fpsyg.2018.01857)
Supplement: Supplementary file 1 [file Data_Sheet_1.PDF]

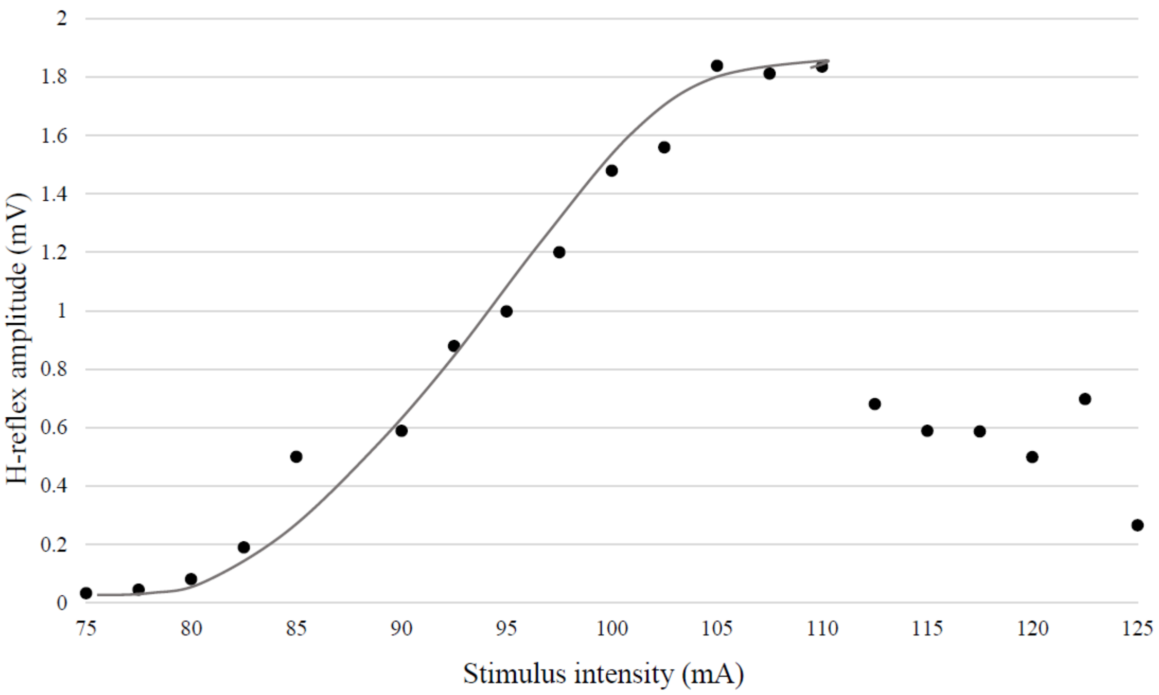

Supp Figure A: example of participant H-reflex responses plotted as a recruitment curve. The H-max is visible at stimulation intensities of 105-110 mA.

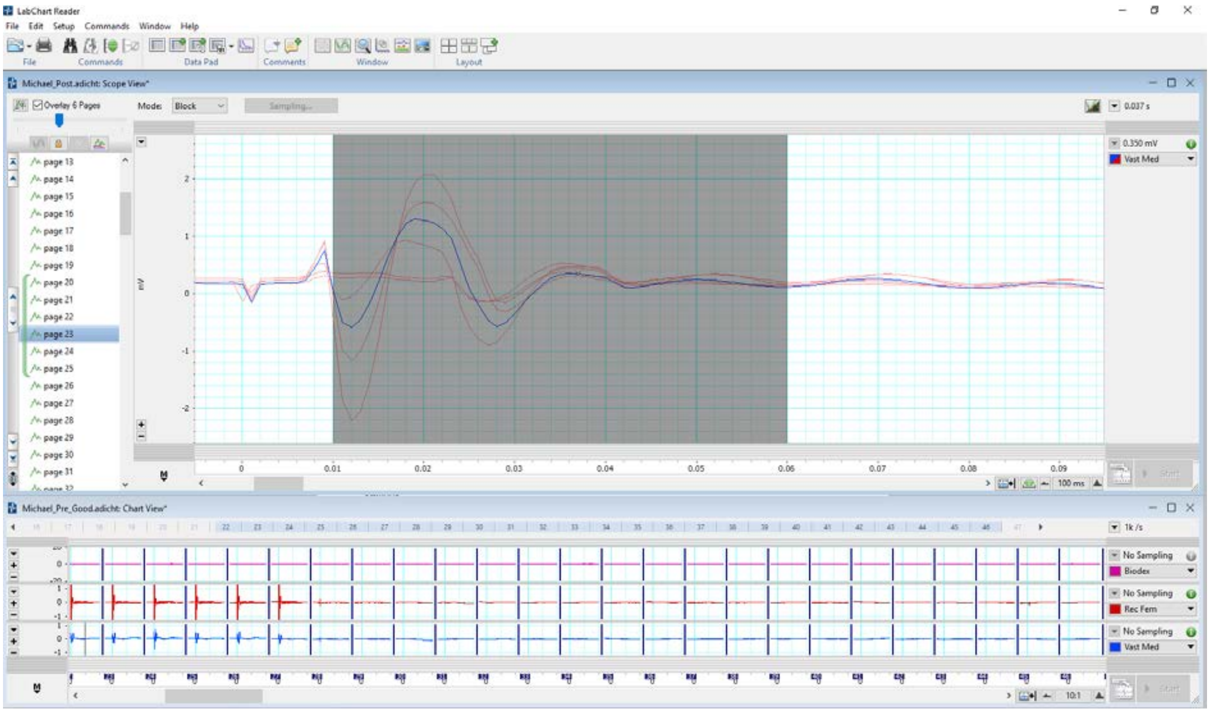

Supp figure B. Example screenshot of raw data showing graded increments of femoral nerve stimulation resulting in M-wave (~10-22ms following stimulus) and H-reflex (~22-40ms following stimulus).
